# Privacy-preserving chi-squared test of independence for small samples

**DOI:** 10.1186/s13040-021-00238-x

**Published:** 2021-01-22

**Authors:** Yuichi Sei, Akihiko Ohsuga

**Affiliations:** The University of Electro-Communications, Tokyo, Japan

**Keywords:** Differentical privacy, Chi-squared testing, Privacy-preserving data mining

## Abstract

**Background:**

The importance of privacy protection in analyses of personal data, such as genome-wide association studies (GWAS), has grown in recent years. GWAS focuses on identifying single-nucleotide polymorphisms (SNPs) associated with certain diseases such as cancer and diabetes, and the chi-squared (*χ*^2^) hypothesis test of independence can be utilized for this identification. However, recent studies have shown that publishing the results of *χ*^2^ tests of SNPs or personal data could lead to privacy violations. Several studies have proposed anonymization methods for *χ*^2^ testing with *ε*-differential privacy, which is the cryptographic community’s de facto privacy metric. However, existing methods can only be applied to 2×2 or 2×3 contingency tables, otherwise their accuracy is low for small numbers of samples. It is difficult to collect numerous high-sensitive samples in many cases such as COVID-19 analysis in its early propagation stage.

**Results:**

We propose a novel anonymization method (RandChiDist), which anonymizes *χ*^2^ testing for small samples. We prove that RandChiDist satisfies differential privacy. We also experimentally evaluate its analysis using synthetic datasets and real two genomic datasets. RandChiDist achieved the least number of Type II errors among existing and baseline methods that can control the ratio of Type I errors.

**Conclusions:**

We propose a new differentially private method, named RandChiDist, for anonymizing *χ*^2^ values for an *I*×*J* contingency table with a small number of samples. The experimental results show that RandChiDist outperforms existing methods for small numbers of samples.

## Introduction

Examining genes involves comparing several groups of genes [1, 2], with three or more groups possibly involved in several instances. Generally, statistical analyses such as the chi-squared (*χ*^2^) test of independence are used to determine whether single-nucleotide polymorphisms (SNPs) can be considered significantly different. The findings from such analyses are frequently shared between researchers and government agencies to facilitate new discoveries.

A genome can contain sensitive information about an individual such as genetic disease factors and disease risk. Each person’s genome is 99.9% identical, with the remaining 0.1% difference producing peoples’ various characteristics. The variation among individuals at a single position in a genome is known as a SNP. A genome-wide association study (GWAS) is a method of analyzing the statistical relationship between SNPs and diseases by finding SNPs that are related to a specific disease. To accomplish this, *χ*^2^ testing has been used. Homer et al. [3] reported that an attacker may be able to statistically determine whether someone is a member of a group with a specific disease if the attacker is familiar with the potential victim’s SNPs and the aggregate allele frequencies within that specific disease group.

The underlying assumption that the attacker is familiar with the potential victim’s SNPs, which can be obtained from a very small blood sample, is realistic because of the increasing availability in cost-effective genotyping services [4, 5]. Furthermore, Wang et al. [6] suggested that the allele frequency of the group SNP values can be determined from standard statistical data such as p-values or *χ*^2^ values. Consequently, an anonymization procedure should always be applied to *χ*^2^ values when publishing SNP datasets [6–8].

Data sharing in genemic research is very important [9]. To avoid such leakage of private information, we should execute a privacy protection mechanism on GWAS results. Existing studies add a relatively large amount of noise to GWAS results to protect privacy. However, our aim is to reduce the amount of noise while maintaining the same level of privacy protection. In other words, we can achieve the same level of privacy protection as existing studies with privacy-preserving *χ*^2^ testing and increase the usefulness of GWAS results.

The recent GWAS analysis methods are not limited to only the chi-squared test. For example, mixed linear model based methods have been used. However, the chi-squared test is still an important analysis method.

Although other methods for GWAS exist, a lot of recent research papers employ the chi-squared test for GWAS, such as [10–13], which were published in 2019 or 2020. Furthermore, the chi-squared test is used in numerous papers on GWAS to analyze COVID-19 [14–18]. Thus, because the chi-squared test has been adopted in many cases, it is worth studying.

Other tests, such as Kruskal-Wallis test and Wilcoxon test, are also employed for GWAS [19, 20]. Couch et al. [21] proposed differentially private methods for these tests. Dealing with other tests in our research remains an issue to be addressed in future work.

The most influential privacy metric within the privacy community is *ε*-differential privacy [22], which has been intensively investigated [23–26]. Several researchers, such as Fienberg et al. [27], Uhlerop et al. [28], and Yu et al. [7], have suggested approaches to facilitate sharing of *χ*^2^ values while conforming with *ε*-differential privacy parameters. However, these proposed methods are currently only applicable to 2×2 or 2×3 contingency tables. In other words, it is currently not possible to analyze contingency tables larger than 2×3. However, the requirement to analyze SNPs based on an *I*×*J* contingency table is crucial. For example, previous studies have evaluated higher degrees of freedom within a contingency table [8, 29]. However, these methods have relatively poor accuracy, particularly in cases with small sample populations. This condition applies to many situations where the sample sizes being considered can range from dozens to several hundred samples [30–32].

Although we live in an era of big data where datasets with a large number of samples are becoming available in many domains, obtaining sensitive information is still difficult due to privacy regulations such as General Data Protection Regulations (GDPR). Sensitive patient biomedical data cannot be shared without permission [33]. Moreover, there are a lot of rare diseases, and obtaining such information of the patients is very difficult [34, 35]. Further, it is difficult to collect a large number of samples when there is a need for rapid analysis for a new disease such as COVID-19. Someone might provide his or her sensitive information without any privatization schemes; however, more people would provide their sensitive information by conducting privatization schemes [36, 37]. Moreover, many studies [38–40] have considered contingency tables larger than 2 ×3. Therefore, private *χ*^2^ testing for large contingency tables with small samples is an important problem.

In this paper, we propose a new method, named RandChiDist, for anonymizing *χ*^2^ values for an *I*×*J* contingency table with a small number of samples, and we experimentally evaluate this method using real datasets. RandChiDist adds the minimized Laplace noise to the true *χ*^2^ value based on the contingency table and controls the ratio of Type I errors (i.e., false positives). The evaluation uses the synthetic and real datasets, including two genomic datasets. The evaluation shows that RandChiDist can control the ratio of Type I errors strictly and can reduce Type II errors (i.e., false negatives) more than existing methods that can control the ratio of Type I errors. Several methods reduce Type II errors more than RandChiDist; however, the methods cannot control the ratio of Type I errors.

Several approaches exist for non-private *χ*^2^ testing, and RandChiDist can be used to calculate the global sensitivity of the *χ*^2^ value of the simplest *χ*^2^ testing and to add noise to the *χ*^2^ value based on the global sensitivity. Thus, the added noise is minimized according to the Laplace mechanism theorem [22].

The motivation of this paper is summarized as follows. Chi-squared test can be employed for various data analyses, such as the identification of SNPs associated with certain diseases; however, publishing the chi-squared value can lead to privacy leakage. Thus, we propose a privacy-preserving chi-squared testing algorithm for a small number of samples due to the difficulty in collecting a large number of samples of a rare disease or new disease.

In our research, samples of less than about 1,000 in number are considered as a small sample size.

The rest of this paper is organized as follows: “Preliminaries” section introduces *χ*^2^ hypothesis test and differential privacy. “Related work” section discusses related work. “Proposed method” section presents our proposed method and “Evaluation” section presents the results of our simulations. “Discussion” Section discusses the evaluation results, and the need for adaptation to a large contingency table and a small sample. “Conclusion” section concludes the paper.

## Preliminaries

### *χ*^2^ hypothesis test of Independence

We consider a contingency table with *I* rows and *J* columns. Let [*i*,*j*] denote the *i*th row and *j*th column’s cell of the table. *O*_*i*,*j*_ represents the value of cell [*i*,*j*], and *E*_*i*,*j*_ represents the expected value of cell [*i*,*j*].

Let $m_{i} \,=\,\! \sum _{j} O_{i,j}, s_{j} \,=\,\! \sum _{i}\! O_{i,j}$, and $n \,=\,\! \sum _{i}\! m_{i} \,=\, \sum _{j} s_{j}$. Table 1 provides an example of a contingency table.

**Table 1:** An example of case-control analysis

	(Combination of allele types of SNP1 and SNP2)				
	(major,major)	(major,minor)	(minor,major)	(minor,minor)	Total
Case	*O*_1,1_	*O*_1,2_	*O*_1,3_	*O*_1,4_	*m*_1_
Control	*O*_2,1_	*O*_2,2_	*O*_2,3_	*O*_2,4_	*m*_2_
Total	*s*_1_	*s*_2_	*s*_3_	*s*_4_	*n*

The *χ*^2^ value is calculated as

1 $$ \begin{aligned} &\chi^{2} \,=\, \sum_{i=1}^{I} \sum_{j=1}^{J} V_{i,j},\\ &\mathrm{where\,\,\,} V_{i,j} = \frac{(E_{i,j} \,-\, O_{i,j})^{2}}{E_{i,j}}, \,\,\,\,E_{i,j} = s_{j} \cdot \frac{m_{i}}{n}.  \end{aligned}  $$

We determine the significance level *α* (i.e., the probability of a Type I error occurring) and the null hypothesis *H*_0_ in advance. We then calculate *χ*^2^ based on Eq. (1) and determine whether to reject *H*_0_ using the *χ*^2^ *distribution table*. Thus, $\chi _{v}^{2}$ represents the probability density function of the *χ*^2^ distribution with *v* degrees of freedom. The *χ*^2^ distribution table presents the percentage point $P(\chi _{v}^{2} > x) = \alpha $ for several combinations of *v* and *α*.

### Privacy model

In recent years, *ε*-differential privacy [22] has been considered the de facto standard for privacy metrics [33, 41–43].

The privacy parameter *ε* reflects the privacy level, with a large *ε* value indicating a low privacy. We consider neighboring databases to represent two databases differing by a maximum of one record. The *ε*-differential privacy is defined as follows:

#### **Definition 1**

(*ε*-differential privacy) Let *D* and *D*^′^ be neighboring databases. A randomized mechanism $\mathcal {M}$ satisfies the *ε*-differential privacy if, for any *D* and *D*^′^ and any subset of outputs $Y \subset Range(\mathcal {M})$, it holds that

2 $$ P(\mathcal{M}(D) \in Y) \le e^{\epsilon} P(\mathcal{M}(D') \in Y).  $$

The Laplace mechanism, which adds noise generated using a Laplace distribution, can satisfy Theorem 1 [22]. To explain this mechanism, we first outline the concept of global sensitivity.

#### **Definition 2**

(Global sensitivity) Let *f* be a function $f : \mathcal {D} \to \mathbb {R}^{d}$, where $\mathcal {D}$ is a collection of databases. When *f* satisfies for any neighoring databases *D* and *D*^′^

3 $$ \Delta f = \max_{D, D'}||f(D) - f(D')||_{1},   $$

the global sensitivity of *f* is *Δf*.

#### **Theorem 1**

(Laplace Mechanism [22]) A randomized mechanism $\mathcal {M}$ realizes *ε*-differential privacy if $\mathcal {M}$ outputs *f*(*D*)+*Lap*(*Δf*/*ε*), where *Lap*(*v*) returns independent Laplace random variables with scale parameter *v*.

## Related work

In *χ*^2^ testing, a contingency table such as Table 2 is used. This contingency table can be represented as Table 3, and Tables 2 and 3 are equivalent. In research on privacy-preserving *χ*^2^ testing, databases such as those shown in Table 3 are considered. For example, Tables 3 and 4 are neighboring databases because the tables contain the same data with exception of one record

**Table 2:** A contingency table

	Condition 1	Condition 2
Group 1	25	30
Group 2	20	25

**Table 3:** A raw database

Pseudo ID	Group number & Condition number
1	1&2
2	1&1
3	2&2
...	...
100	2&1

**Table 4:** Database that contains the same data as Table 3 except with 3’s data

Pseudo ID	Group number & Condition number
1	1&2
2	1&1
3’	2&1
...	...
100	2&1

Yu et al. [7] demonstrated that the global sensitivity of the *χ*^2^ value of 2×3 contingency tables can be calculated as

4 $$ \Delta_{Y} = \frac{n^{2}}{m_{1} m_{2}}\left(1-\frac{1}{\max{\{m_{1}, m_{2}\}}+1}\right),   $$

if *m*_1_ and *m*_2_ are known (i.e., published).

Fienberg et al. [27] and Uhlerop et al. [28] demonstrated that if *m*_1_=*m*_2_, the global sensitivity of the *χ*^2^ value can be calculated as

5 $$ \Delta_{F} = \frac{4n}{n+2}.   $$

The global sensitivities, *Δ*_*F*_ and *Δ*_*Y*_, have been shown to be optimal values. However, they can only be applied to 2×2 or 2×3 contingency tables.

Kakizaki et al. [44, 45] proposed a unit circle mechanism that can achieve a high degree of accuracy. However, they assumed only 2×2 contingency tables. Additionally, they did not publish the differentially private *χ*^2^ value used in their method; however, they did publish the differentially private result of the *χ*^2^ testing based on the given significance level, *α*. Therefore, if a data holder wants to publish the private *χ*^2^ testing results of several *α* values (e.g., *α*=0.05,0.01,0.005, and 0.001), the data holder must independently execute the privacy mechanisms multiple times (e.g., three times). Following the composition theorem [46], if a privacy mechanism outputs *K* times based on *ε*-differential privacy, the resulting privacy level thus becomes *Kε* (i.e., the privacy level decreases). Moreover, Banerjee et al. [47] state that publishing P-value could be important for data analysis.

The aforementioned studies all assumed that *m*_*i*_ (*i*=1,…,*I*) is not sensitive information. We can share each value of *m*_*i*_ without privatization schemes.

Gaboardi et al. [8] proposed several methods for arbitrary contingency tables. First, they show a straightforward method that does not add Laplace noise to the *χ*^2^ value, but rather adds it to each cell of the contingency table with a global sensitivity of 2. In this paper, we name this method as RandCell. RandCell is also known as SNPpval, which was proposed by Jonson and Shmatikov [48]. The *χ*^2^ value of the contingency table to which RandCell adds Laplace noise tends to be large, meaning that RandCell yields many false positives. Therefore, Gaboardi et al. proposed several other methods known as PrivIndep, MCIndep with Laplace mechanism, and MCIndep with Gaussian mechanism. They showed that MCIndep with Laplace mechanism had the best performance of their proposed methods. Hence, we describe MCIndep with Laplace mechanism in detail in this paper and refer to MCIndep with Laplace mechanism as MCIndep for simplicity.

MCIndep generates many contingency tables randomly based on *m*_*i*_ and *s*_*j*_ of the contingency table with added Laplace noise and compares their *χ*^2^ values. The original contingency table can be considered to reject *H*_0_ if the *χ*^2^ value of the contingency table to which RandCell adds Laplace noise is greater than the top *α*×100*%* of the generated contingency tables’ *χ*^2^ value. Other methods for (*ε*,*δ*)-differential privacy are proposed [49], which relaxes the *ε*-differential privacy as their privacy metric. We focus on *ε*-differential privacy in this paper, and applying our method to (*ε*,*δ*)-differential privacy is an issue to be addressed in future work.

Sei et al. [50] proposed several theorems for differentially private *χ*^2^ testing, but there were no detailed proofs for the theorems and the equations provided in their study. Moreover, there were no experiments that evaluated the performance of *χ*^2^ testing.

More recently, Gaboardi et al. [29] proposed *χ*^2^ test algorithms (LocalNoiseIND, LocalExpIND, and LocalBitFlipIND) for privacy-preserving *χ*^2^ testing of independence based on local differential privacy. LocalNoiseIND is also known as zCDP general chi-squared test, which was proposed by Kifer and Rogers [51]. In their paper, they showed that LocalExpIND had the best performance of the three methods for most parameter settings. These methods can be applied to arbitrary contingency tables, and address a local model of privacy and assume there is no trusted entity. In this paper, we assume that a trusted entity has all the raw data.

Canonne et al. [52] calculated the sample complexity bounds of an *ε*-differentially private test for distinguishing between two distributions. They also applied differentially private change-point detection. Their method is for a parametric setting that requires that the two distributions are perfectly known. In contrast, our method can be used for a nonparametric setting.

Csail et al. [53] proposed an algorithm for testing the closeness of two distributions in a private manner. Their algorithm can also test the independence of two random variables. However, execution for privacy-preserving *χ*^2^ testing was not described.

Liu et al. [54] showed how *ε* influences the accuracy of differentially private hypothesis testing. They proposed a method to determine an appropriate value for *ε* that can be useful for determining the *ε* value for our proposed algorithm; however, determining *ε* is outside the scope of our paper.

Couch et al. [21] proposed a differentially private hypothesis testing method for the Kruskal-Wallis test, Mann-Whitney test, Wilcoxon test, and one-sample t-test. This hypothesis testing method is not for nominal scale data, which are suitable for *χ*^2^ testing, but rather for ordinal or interval scale data.

The methods for arbitrary *I*×*J* contingency tables have relatively poor accuracy, particularly in cases with small-sample populations. We show the comparison between existing methods and the proposed method in “Evaluation” section.

### Adversarial model

The adversarial model is described as follows. The server has a database, and it wants to share the result of the chi-squared test with data analysts who are potential attackers. The attacker is considered to be a semi-honest entity, that is, the attacker follows the protocol between the server. However, the attacker might attempt to extract individual information from the result of the chi-squared test.

## Proposed method

### Overview

We propose RandChiDist, which adds Laplace noise to the *χ*^2^ value obtained from a target contingency table. Calculating the Laplace noise to be added requires the global sensitivity of the *I*×*J* contingency table’s *χ*^2^ value. The method for calculating global sensitivity is described in 1.

Typically, the *χ*^2^ distribution table is used to determine whether to reject *H*_0_. However, RandChiDist adds noise to the *χ*^2^ value, thus we need a modified *χ*^2^ distribution table. The method for calculating this is described in 1. RandChiDist uses this table to determine whether to reject *H*_0_. We consider bounding the Type I error to be at most *α* to be a hard constraint.

Our main symbols are summarized in Table 5.

**Table 5:** Symbols

*n*	Number of samples
*I*	Number of rows of a table
*J*	Number of columns of a table
*m*_*i*_	Total value of *i*th row
*s*_*j*_	Total value of *j*th column
*O*_*i*,*j*_(*D*)	Observed value of cell [*i*,*j*] in database *D*
*E*_*i*,*j*_(*D*)	Expected value of cell [*i*,*j*] in database *D*

### Global sensitivity of *χ*^2^ value

As was assumed in other studies, we assume that *m*_*i*_ (*i*=1,…,*I*) is also provided to a data analyzer. We consider contingency tables *D*_1_ and *D*_2_, which are generated from neighboring databases. Because the neighboring databases differ by one record, their contingency tables differ by a maximum of two cells. The value of cell [*a*,*k*] in table *D*_2_ is greater than that of cell [*a*,*k*] in *D*_1_ by 1, and the value of cell [*a*,*l*] in table *D*_2_ is less than that of cell [*a*,*l*] (s.t. *l*≠*k*) in table *D*_1_ by 1.

Because the values of *m*_*i*_(*i*=1,…,*I*) are released to the public, the collection of databases in Definition 2.2 only include databases that satisfy the released values of *m*_*i*_, and the neighboring databases are elements of the collection. Therefore, the global sensitivity is calculated based on the neighboring databases that satisfy the released values of *m*_*i*_.

Thus, we calculate the possible maximum value of the difference of *χ*^2^ values between tables *D*_1_ and *D*_2_.

RandChiDist satisfies differential privacy by adding Laplace noise with global sensitivity because of Theorem 1. We thus propose RandChiDist, which adds Laplace noise with global sensitivity,

6 $$ \Delta_{R} = \left\{\begin{array}{ll} \frac{(m_{\alpha}+m_{\beta})n}{m_{\alpha}(1+m_{\beta})} & J\ge 3\\ \frac{n^{2}}{m_{\alpha} (n-m_{\alpha}+1)} & J=2, \end{array}\right.  $$

where

7 $$ \begin{aligned} &\alpha = \underset{i}{\text{arg min}}\ {m_{i}} \mathrm{\,\,\,\,\,\,and}\\ &\beta = \underset{i\neq \alpha}{\text{arg min}}\ {m_{i}}, \end{aligned}  $$

to the calculated *χ*^2^ value from (1). Here, we have the following theorem:

#### **Theorem 2**

RandChiDist satisfies *ε*-differential privacy.

#### *Proof*

We prove that *Δ*_*R*_ is the global sensitivity of *χ*^2^ of the *I*×*J* contingency table. We can then uphold Theorem 2 because RandChiDist adds *Lap*(*Δ*_*R*_/*ε*) to the original value based on the Laplace mechanism theorem (Theorem 1).

Let *O*_*i*,*j*_(*D*) denote the observed value of cell [*i*,*j*] in database *D* and let *χ*^2^(*D*) denote the *χ*^2^ value of database *D*. Without a loss of generality, we consider neighboring databases *D*_1_ and *D*_2_, which satisfy the following equations:

8 $$ \left\{\begin{array}{l} O_{a,k}(D_{2}) = O_{a,k}(D_{1})+1 \\ O_{a,l}(D_{2}) = O_{a,l}(D_{1})-1, \end{array}\right.  $$

where *k* and *l* are arbitrary natural numbers satisfying *k*,*l*∈{1,…,*J*} and *k*≠*l*.

**Fig. 1:**
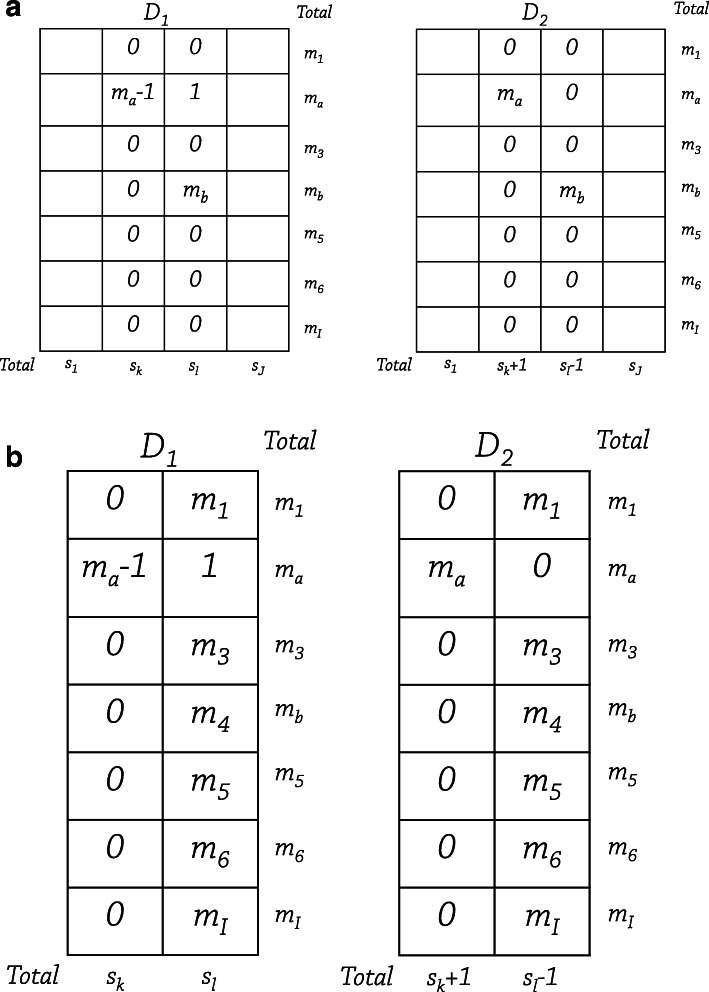
Neighboring databases. **a** Tables for *J*≥3. **b** Tables for *J*=2

From Proposition 2, when *J* is greater than or equal to 3 and we are given the value *a*, neighboring databases that satisfy the following constraints maximize the difference between the *χ*^2^ values of tables *D*_1_ and *D*_2_ (see Fig. 1a).

9 $$ \begin{aligned} O_{a,k}(D_{1}) +1 &= O_{a,k}(D_{2}) = m_{a}\\ O_{a,l}(D_{1})-1 &= O_{a,l}(D_{2}) = 0\\ O_{b,l}(D_{1}) &= O_{b,l}(D_{2}) = m_{b} \mathrm{\,\,where\,\,} b\neq a\\ O_{i,k}(D_{1}) & =0 \mathrm{\,\, where\,\,} i\neq a\\ O_{i,l}(D_{1}) & =0 \mathrm{\,\, where\,\,} i\neq a,b\\ O_{i,j}(D_{1}) &= O_{i,j}(D_{2}) = \mathrm{arbitrary\,\,values\,\,that}\\ &\mathrm{satisfy\,\,the\,\,constraint\,\,} \sum_{j} O_{i,j} = m_{i} \\ &\mathrm{where\,\,} [i,j] \neq [a,k], [a,l], \mathrm{\,and\,} [b,l]. \end{aligned}  $$

From constraint (9), we understand that the sum of the kth column of *D*_2_ (i.e., *s*_*k*_ of *D*_2_) is equal to *m*_*a*_.

Let *V*_*i*,*j*_(*D*) denote *V*_*i*,*j*_ in Eq. (1) for database *D*.

The symbol *b* is an arbitrary integer from 1 to *I* but not a. The symbol *l* is an arbitrary integer from 1 to *J* but not *k*.

The difference between the *χ*^2^ values of tables *D*_1_ and *D*_2_ that satisfies the constraint (9) is thus calculated by

10 $$ {}\begin{aligned} &\sum_{i} (V_{i,k}(D_{2}) + V_{i,l}(D_{2}) - V_{i,k}(D_{1}) - V_{i,l}(D_{1}))=V_{a,k}(D_{2}) + \sum_{i\neq a} V_{i,k}(D_{2}) + V_{b,l}(D_{2})\\ &\quad+ \sum_{i\neq b}V_{i,l}(D_{2}) -V_{a,k}(D_{1}) - \sum_{i\neq a} V_{i,k}(D_{1}) - V_{a,l}(D_{1}) - V_{b,l}(D_{1})\\ &\quad- \sum_{i\neq a, b}V_{i,l}(D_{1}) =\frac{(m_{a} \frac{m_{a}}{n} - m_{a})^{2}}{m_{a} \frac{m_{a}}{n}}+\sum_{i\neq a} \frac{(m_{a} \frac{m_{i}}{n})^{2}}{m_{a} \frac{m_{i}}{n}} +\frac{(m_{b} \frac{m_{b}}{n} - m_{b})^{2}}{m_{b} \frac{m_{b}}{n}}+\sum_{i\neq b} \frac{(m_{b} \frac{m_{i}}{n})^{2}}{m_{b} \frac{m_{i}}{n}}\\ &\quad-\frac{((m_{a}-1) \frac{m_{a}}{n} - (m_{a}-1))^{2}}{(m_{a}-1) \frac{m_{a}}{n}}-\sum_{i\neq a} \frac{((m_{a}-1) \frac{m_{i}}{n})^{2}}{(m_{a}-1) \frac{m_{i}}{n}} -\frac{((m_{b}+1) \frac{m_{a}}{n} - 1)^{2}}{(m_{b}+1) \frac{m_{a}}{n}}\\ &\quad-\frac{((m_{b}+1) \frac{m_{b}}{n} - m_{b})^{2}}{(m_{b}+1) \frac{m_{b}}{n}} -\sum_{i\neq a,b} \frac{((m_{b}+1) \frac{m_{i}}{n})^{2}}{(m_{b}+1) \frac{m_{i}}{n}} =\frac{(m_{a} + m_{b})n}{m_{a}(1+m_{b})} \end{aligned}  $$

Therefore, given *a*, when the value of *J* is greater than or equal to 3, global sensitivity is represented by Eq. (10). Moreover, from Proposition 1, global sensitivity is represented by Eq. (6) when the value of *J* is greater than or equal to 3 and *a* is not given.

When *J*=2 and *a* is given, neighboring databases that satisfy the following constraints will maximize the difference between the *χ*^2^ values of contingency tables *D*_1_ and *D*_2_ from Proposition 3 (see Fig. 1b).

11 $$ \begin{aligned} O_{a,k}(D_{1}) +1 &= O_{a,k}(D_{2}) = m_{a}\\ O_{a,l}(D_{1})-1 &= O_{a,l}(D_{2}) = 0\\ O_{i,k}(D_{1}) &= O_{i,k}(D_{2}) = 0 \mathrm{\,\,for\,\,all\,\,}i\mathrm{\,\,except\,\,for\,\,}i=a\\ O_{i,l}(D_{1}) &= O_{i,l}(D_{2}) = m_{i} \mathrm{\,\,for\,\,all\,\,}i\mathrm{\,\,except\,\,for\,\,}i=a \end{aligned}  $$

The difference between the *χ*^2^ values of tables *D*_1_ and *D*_2_ that satisfy the constraint (11) can be calculated as

12 $$ {}\begin{aligned} &\sum_{i} (V_{i,k}(D_{2}) + V_{i,l}(D_{2}) - V_{i,k}(D_{1}) - V_{i,l}(D_{1})) =V_{a,k}(D_{2}) + \sum_{i\neq a} V_{i,k}(D_{2}) + V_{a,l}(D_{2}) \\ &\quad+ \sum_{i\neq a}V_{i,l}(D_{2}) -V_{a,k}(D_{1}) - \sum_{i\neq a} V_{i,k}(D_{1}) - V_{a,l}(D_{1}) - \sum_{i\neq a}V_{i,l}(D_{1}) =\frac{(m_{a} \frac{m_{a}}{n} - m_{a})^{2}}{m_{a} \frac{m_{a}}{n}}\\ &\quad+ \sum_{i\neq a} \frac{(m_{a} \frac{m_{i}}{n})^{2}}{m_{a} \frac{m_{i}}{n}} +\frac{((n-m_{a}) \frac{m_{a}}{n})^{2}}{(n-m_{a}) \frac{m_{a}}{n}} + \sum_{i\neq a} \frac{((n-m_{a}) \frac{m_{i}}{n} - m_{i})^{2}}{(n-m_{a}) \frac{m_{i}}{n}}\\ &\quad-\frac{((m_{a}-1) \frac{m_{a}}{n} - (m_{a}-1))^{2}}{(m_{a}-1) \frac{m_{a}}{n}}-\sum_{i\neq a} \frac{((m_{a}-1) \frac{m_{i}}{n})^{2}}{(m_{a}-1) \frac{m_{i}}{n}} -\frac{((n-m_{a}+1) \frac{m_{a}}{n} - 1)^{2}}{(n-m_{a}+1) \frac{m_{a}}{n}}\\ &\quad-\sum_{i\neq a} \frac{((n-m_{a}+1) \frac{m_{i}}{n} - m_{i})^{2}}{(n-m_{a}+1) \frac{m_{i}}{n}} =\frac{n^{2}}{m_{a}(n-m_{a}+1)}. \end{aligned}  $$

Because *n*^2^/(*m*_*a*_(*n*−*m*_*a*_+1)) decreases when *m*_*a*_ decreases, the global sensitivity can be represented by Eq. (6) when *J* is equal to 2 and *a* is not given. □

When we use a 2×3 contingency table, *Δ*_*R*_ is identical to *Δ*_*Y*_, and when we use a 2×3 contingency table with *m*_1_=*m*_2_,*Δ*_*R*_ is identical *Δ*_*F*_.

Propositions 1 and 2 used in the proof of Theorem 2 are described below.

#### **Proposition 1**

*Δ*_*R*_ in Eq. (6) is maximized when the minima (7) are satisfied.

#### *Proof*

By differentiating Eq. (6) with respect to *m*_*a*_, we obtain

13 $$ -\frac{m_{b} n}{m_{a}^{2} (1+m_{b})}.   $$

By differentiating Eq. (6) with respect to *m*_*b*_, we obtain

14 $$ -\frac{(m_{a} - 1)n}{m_{a} (1+m_{b})^{2}}.   $$

From Expressions (13) and (14), *m*_*a*_ and *m*_*b*_ should thus be minimized to maximize Eq. (6).

Let *min* denote the minimum value in *m*_*i*_ (*i*=1,…,*I*) and let *min*+*x* denote the second most minimum value in *m*_*i*_ (*i*=1,…,*I*), where *x*≥0. If *m*_*a*_ is *min* and *m*_*b*_ is *min*+*x*, Eq. (6) can then be expressed as

15 $$ \frac{n(2min+x)}{min(1+min+x)}.  $$

If *m*_*a*_ is *min*+*x* and *m*_*b*_ is *min*, Eq. (6) can then be expressed as

16 $$ \frac{n(2min+x)}{(1+min)(min+x)}.  $$

Because Expression (15) is always greater than or equal to Expression (16), we find that the value of *Δ*_*R*_ in Eq. (6) is maximized when (7) is satisfied. □

#### **Proposition 2**

When *J* is greater than or equal to 3 and *a* is given, neighboring databases that satisfy the constraints (9) maximize the difference between the *χ*^2^ values of tables *D*_1_ and *D*_2_.

#### *Proof*

There are many neighboring databases that satisfy Eq. (8); however, we prove that neighboring databases that satisfy the constraints (9) have the greatest difference, *δ*(*D*_1_,*D*_2_), between *χ*^2^(*D*_1_) and *χ*^2^(*D*_2_) when *J*≥3. We assume that *m*_*i*_ is a fixed value for all values of *i*.

Thus, we write *O*_*i*,*j*_(*D*_1_) as *O*_*i*,*j*_ for any *i* and *j* in the following manner.

Following Lemma 1, *O*_*a*,*k*_ should be maximized to maximize *δ*(*D*_1_,*D*_2_). As a result, the value of *O*_*a*,*k*_ becomes *m*_*a*_−1 because of the constraints (17).

From Eq. (8) we have the following constraints:

17 $$ O_{a,k}(D_{1}) \le m_{a}-1\,\,\,\,\,\text{and}\,\,\,\,\,1 \le O_{a,l}.   $$

Following Lemma 2, *O*_*i*,*k*_ should be zero to maximize *δ*(*D*_1_,*D*_2_) for all values of *i* except *i*=*a*.

Following Lemma 3, *O*_*a*,*l*_ should be minimized to maximize *δ*(*D*_1_,*D*_2_). As a result, the value of *O*_*a*,*l*_ becomes 1 because of the constraints (17).

Following Lemma 4, *O*_*μ*,*l*_*μ*≠*a* should be *m*_*μ*_ and *O*_*i*,*l*_ for all *i*, except for *i*=*a*, and *i*=*μ* should be zero to maximize *δ*(*D*_1_,*D*_2_).

As a result, we can maximize *δ*(*D*_1_,*D*_2_) when tables *D*_1_ and *D*_2_ satisfy the constraints (9) by replacing *μ* in Lemma 4 with *b*. □

#### **Lemma 1**

To maximize *δ*(*D*_1_,*D*_2_),*O*_*a*,*k*_ should be maximized (and correspondingly, *O*_*a*,*r*_ for all *r*, except for *r*=*k*,*l*, should be adjusted to satisfy *m*_*a*_).

#### *Proof*

We have

18 $$ \begin{aligned} &\delta(D_{1}, D_{2}) = \chi^{2}(D_{2}) - \chi^{2}(D_{1}) \\ &= V_{a,k}(D_{2})-V_{a,k}(D_{1}) + \sum_{i\neq a} (V_{i,k}(D_{2}) - V_{i,k}(D_{1})) \\ &+ V_{a,l}(D_{2})-V_{a,l}(D_{1}) + \sum_{i\neq a} (V_{i,l}(D_{2}) - V_{i,l}(D_{1}))\\ &= -2+ \frac{m_{a}}{n} +\frac{n(-O_{a,k}^{2} + s_{k} + 2s_{k} O_{a,k})}{m_{a}s_{k}(1 + s_{k})} \\ &+ \sum_{i\neq a} \frac{m_{i}^{2} s_{k} (1+s_{k}) - n^{2} O_{i,k}^{2}}{m_{i} n s_{k} (1+s_{k})}\\ &+2 - \frac{m_{a}}{n} + \frac{n(O_{a,l}^{2} + s_{l} - 2s_{l} O_{a,l})}{m_{a}(s_{l}-1)s_{l}}\\ &+ \sum_{i\neq a} \frac{n^{2} O_{i,l}^{2} - m_{i}^{2} (s_{l}-1) s_{l}}{m_{i} n (s_{l}-1)s_{l}}. \end{aligned}  $$

By differentiating Eq. (18) with respect to *O*_*a*,*k*_, we obtain

19 $$ \frac{n(O_{a,k}-s_{k})^{2}(1+2s_{k})}{m_{a} s_{k}^{2} (1+s_{k})^{2}}+\sum_{i\neq a} \frac{n O_{i,k}^{2} (1+2s_{k})}{m_{i} s_{k}^{2} (1+s_{k})^{2}},   $$

because we have

20 $$ \frac{\partial s_{k}}{\partial O_{a,k}} = 1.  $$

Because Expression (19) is always ≥0, Eq. (18) increases as *O*_*a*,*k*_ increases.

Thus, we can say that *O*_*a*,*k*_ should be increased to maximize *δ*(*D*_1_,*D*_2_). As a result, we have *O*_*a*,*k*_=*m*_*a*_−1. □

#### **Lemma 2**

To maximize *δ*(*D*_1_,*D*_2_),*O*_*i*,*k*_ should be minimized (and correspondingly *O*_*i*,*r*_ for all *r*, except for *r*=*k*,*l*, should be adjusted to satisfy *m*_*i*_) for all values of *i* except for *i*=*a*.

#### *Proof*

We focus on *μ*∈{1,…,*I*} such that *μ*≠*a*. By differentiating Eq. (18) with respect to *O*_*μ*,*k*_, we obtain

21 $$ \begin{aligned} &\frac{n(O_{a,k}-s_{k})(O_{a,k}+s_{k} + 2s_{k} O_{a,k})}{m_{a} s_{k}^{2}(1+s_{k})^{2}}\\ &+\frac{nO_{\mu,k}(O_{\mu,k}+2s_{k} O_{\mu,k}-2s_{k}(1+s_{k})}{m_{\mu} s_{k}^{2}(1+s_{k})^{2}}\\ &+\sum_{i\neq a, \mu} \frac{n O_{i,k}^{2} (1+2s_{k})}{m_{i} s_{k}^{2} (1+s_{k})^{2}}, \end{aligned}  $$

because we have

22 $$ \frac{\partial s_{k}}{\partial O_{\mu,k}} = 1.  $$

Let $\Theta = \sum _{i\neq a,\mu } O_{i,k}^{2}/m_{i}$. By solving equation (21) =0 for *Θ*, we obtain

23 $$ \begin{aligned} &(m_{\mu} (s_{k}-O_{a,k})(O_{a,k}+s_{k}+2s_{k}O_{a,k})\\ & +m_{a} O_{u,k}(2s_{k}-O_{u,k} + 2s_{k}(s_{k}-O_{u,k}))\\ &/(m_{a} m_{\mu} (1+2s_{k})). \end{aligned}  $$

Expression (23) is always greater than zero. When *Θ*=0 in Expression (21), the value of Expression (21) is less than 0.

Thus, when *Θ* is less than Expression (23), Expression (21) is less than zero. Similarly, when *Θ* is greater than Expression (23), Expression (21) is greater than zero. That is, to maximize Eq. (18), the value of *O*_*μ*,*k*_ should be either minimized or maximized. From this observation, to maximize Eq. (18), we can say that *O*_*i*,*k*_ should be either minimized (i.e., zero) or maximized (i.e., *m*_*i*_) for all *i* except for *i*=*a*,.

From Lemma 1, we have *O*_*a*,*k*_=*m*_*a*_−1. Therefore, when *O*_*i*,*k*_=0 for all *i* except *i*=*a*, we have *s*_*k*_=*m*_*a*_−1. In this case, *δ*(*D*_1_,*D*_2_) is

24 $$ \begin{aligned} &-2 + \frac{m_{a}}{n} + \frac{n}{m_{a}} + \frac{1}{n} \sum_{i\neq a} m_{i} \\ &+ V_{a,l}(D_{2})-V_{a,l}(D_{1}) + \sum_{i\neq a} (V_{i,l}(D_{2}) - V_{i,l}(D_{1})). \end{aligned}  $$

In contrast, when *O*_*i*,*k*_=*m*_*i*_ for all *i* except $i=a, s_{k} = \sum _{i} m_{i}-1 = n-1$. In this case, *δ*(*D*_1_,*D*_2_) is

25 $$ \begin{aligned} &-2+\frac{m_{a}}{n} + \frac{n}{m_{a}}+\sum_{i\neq a}\frac{m_{i}}{n}-\frac{(n-m_{a})^{2}}{m_{a}(n-1)n} + \sum_{i\neq a}\frac{m_{i}}{n-n^{2}}. \end{aligned}  $$

By subtracting Expression (25) from Expression (24), we obtain

26 $$ \frac{(n-m_{a})^{2}}{m_{a}(n-1)} + \sum_{i\neq a} \frac{m_{i}}{n-1}.   $$

Because Expression (26) is always greater than zero, *O*_*i*,*k*_ for all *i* except *i*=*a* should be zero. □

#### **Lemma 3**

To maximize *δ*(*D*_1_,*D*_2_),*O*_*a*,*l*_ should be minimized (and correspondingly *O*_*a*,*r*_ for all *r* except *r*=*k*,*l* should be adjusted to satisfy *m*_*a*_).

#### *Proof*

By differentiating Eq. (18) with respect to *O*_*a*,*l*_, we obtain

27 $$ \frac{n(O_{a,l}-s_{l})^{2}(1-2s_{l})}{m_{a}(s_{l}-1)^{2}s_{l}^{2}} + \sum_{i\neq a}\frac{nO_{i,l}^{2}(1-2s_{l})}{m_{i}(s_{l}-1)^{2}s_{l}^{2}},  $$

because we have

28 $$ \frac{\partial s_{l}}{\partial O_{a,l}} = 1.  $$

Because (27) is always less than zero, Eq. (18) increases as *O*_*a*,*l*_ decreases. □

#### **Lemma 4**

To maximize *δ*(*D*_1_,*D*_2_),*O*_*μ*,*l*_ (*μ*≠*a*) should be maximized (and correspondingly, *O*_*μ*,*r*_ for all *r* except *r*=*k*,*l* should be adjusted to satisfy *m*_*μ*_). Additionally, *O*_*i*,*l*_ should be minimized (and correspondingly, *O*_*i*,*r*_ for all *r* except *r*=*k*,*l* should be adjusted to satisfy *m*_*i*_) for all *i* except *i*=*a* and *i*=*μ*.

#### *Proof*

By differentiating Eq. (18) with respect to *O*_*μ*,*l*_(*μ*≠*a*), we obtain

29 $$ \begin{aligned} &\frac{n(s_{l}-O_{a,l})(2s_{l} O_{a,l}-O_{a,l}-s_{l})}{m_{a}(s_{l}-1)^{2}s_{l}^{2}}\\ &+\frac{n O_{\mu, l} (O_{\mu,l} -2 s_{l} -2 s_{l} O_{\mu,l} + 2s_{l}^{2})}{m_{\mu} (s_{l}-1)^{2} s_{l}^{2}}\\ &+\sum_{i\neq a,\mu} \frac{n O_{i,l}^{2}(1-2s_{l})}{m_{i}(s_{l}-1)^{2}s_{l}^{2}}, \end{aligned}  $$

because we have

30 $$ \frac{\partial s_{l}}{\partial O_{\mu,l}} = 1.  $$

Let $\Theta = \sum _{i\neq a,\mu } O_{i,l}^{2}/m_{i}$. We have *O*_*a*,*l*_=1 from Lemma 3. By solving Expression (29)=0 for *Θ*, we obtain

31 $$ \frac{m_{\mu} (s_{l}-1)^{2} + m_{a} O_{u,l} (2s_{l} (s_{l}-O_{\mu,l}-1)+O_{\mu,l})}{m_{a} m_{\mu} (2s_{l}-1)}.  $$

Expression (31) is always greater than zero. When *Θ*=0 and *O*_*a*,*l*_=1 in Expression (29), (29) can be expressed as

32 $$ \frac{n(m_{\mu} (s_{l}-1)^{2} + m_{a} O_{\mu,l} (2s_{l} (s_{l}-O_{\mu,l}-1) + O_{\mu,l}))}{m_{a} m_{\mu} (s_{l}-1)^{2} s_{l}^{2}} \ge 0.   $$

Therefore, when *Θ* is less than or equal to Expression (31), Expression (29) is greater than zero, and when *Θ* is greater than Expression (31), Expression (29) is less than zero. Thus, to maximize Eq. (18), the value of *O*_*μ*,*l*_ should be either minimized (i.e., zero) or maximized (i.e., *m*_*μ*_).

Thus, to maximize *δ*(*D*_1_,*D*_2_), the value of *O*_*μ*,*l*_ should be either minimized or maximized. Let us have $x = \sum _{i\neq \mu } O_{i,l}$. When *O*_*μ*,*l*_ is maximized (i.e., *O*_*μ*,*l*_=*m*_*μ*_), we *δ*(*D*_1_,*D*_2_) is

33 $$ \begin{aligned} &2-\frac{m_{a} + m_{\mu}}{n}+\frac{m_{\mu} n}{m_{\mu} + x -1} - \frac{n + m_{a} m_{\mu} n}{m_{a} (m_{\mu} + x)}\\ &+\sum_{i\neq a, \mu} \left(\frac{O_{i,l}^{2} n}{m_{i} (m_{\mu} + x-1)(m_{\mu} + x)} - \frac{m_{i}}{n}\right). \end{aligned}  $$

In contrast, when *O*_*μ*,*l*_ is minimized (i.e., *O*_*μ*,*l*_=0), *δ*(*D*_1_,*D*_2_) is

34 $$ \begin{aligned} &2-\frac{m_{a} + m_{\mu}}{n} - \frac{n}{m_{a} x}\\ &+\sum_{i\neq a, \mu} \frac{O_{i,l}^{2} n^{2} - m_{i}^{2} (x-1)x}{m_{i} n (x-1)x}. \end{aligned}  $$

By subtracting Expression (34) from Expression (33), we obtain

35 $$ \begin{aligned} &\frac{m_{\mu} n (-1+m_{\mu} + x + m_{a} x)}{m_{a} x (-1 + m_{\mu} + x)(m_{\mu} + x)}\\ &-\sum_{i\neq a, \mu} \frac{ n m_{\mu} O_{i,l}^{2} (-1 + m_{\mu} + 2x)}{m_{i}(x-1)x(m_{\mu} + x-1)(m_{\mu} + x)}. \end{aligned}  $$

When *I*=2, the second term of Expression (35) is zero. Therefore, Expression (35) is always greater than zero and Lemma 4 holds when *I*=2.

We then consider the situation where *I*≥3. We assume that *O*_*i*,*l*_ is zero for all values of *i* except *i*=*a* and *i*=*μ*. In this case the second term of Expression (35) is zero and the first term of Expression (35) is greater than zero; therefore, we can say that Expression (35) is always greater than zero. Thus, *O*_*μ*,*l*_ should be maximized to *m*_*μ*_ when *O*_*i*,*l*_ is zero for all values of *i* except *i*=*a* and *i*=*μ*.

Next, we focus on *v* such that *v*∈{1,…,*I*} and *v*≠*a*,*μ*. We demonstrate that Expression (35) is always ≤ 0 when *O*_*v*,*l*_ is maximized to *m*_*v*_. Additionally, the second term of Expression (35) is minimized when *I*=3. In this case, we obtain

36 $$ (35) \le - \frac{(m_{a}-1)m_{\mu} n}{m_{a}(1+m_{v})(1+m_{v}+m_{\mu})} < 0,  $$

because *x*=*m*_*v*_+1.

Therefore, each *O*_*i*,*l*_ for all *i* except *i*≠*a*,*μ* should be minimized to zero.

From this observation, Lemma 4 also holds when *I*≥3. □

#### **Proposition 3**

When *J* equals 2 and *a* is given, neighboring databases that satisfy the constraints (11) maximize the difference between the *χ*^2^ values of tables *D*_1_ and *D*_2_.

The proof can be conducted in a similar manner as Lemma 2.

### Differentially private hypothesis testing

We can now calculate the anonymized *χ*^2^ value from an original table,

37 $$ {\chi^{2}}^{*} = \chi^{2} + Lap(\Delta_{R}/\epsilon),   $$

where *χ*^2^^∗^ is the anonymized *χ*^2^ value.

From the definitions of the Laplace distribution and *χ*^2^ distribution, the probability density function of a *χ*^2^ value possessing *v* degrees of freedom with the addition of Laplace noise and global sensitivity *Δ* can be expressed as

38 $$ g_{v, \Delta, \epsilon}(x) = \int_{\mu=-\infty}^{\infty} \mathcal{L}_{\mu,\beta}(x) \mathcal{Z}_{v}(\mu) d\mu,  $$

where

39 $$ \beta = \Delta/\epsilon,  $$

40 $$ \mathcal{L}_{\mu,\beta}(x) = \left\{\begin{array}{ll} \frac{\exp{\left(-\frac{x-\mu}{\beta}\right)}}{2\beta} & x \ge \mu \\ \frac{\exp{\left(-\frac{\mu-x}{\beta}\right)}}{2\beta} & otherwise, \end{array}\right.  $$

and

41 $$ \mathcal{Z}_{v}(u) = \left\{\begin{array}{ll} \frac{2^{-v/2} \exp{(-u/2)} u^{-1+v/2}}{\Gamma(v/2)} & x>0 \\ 0 & otherwise, \end{array}\right.  $$

where *Γ*(*v*/2) represents the *v*/2 gamma function, that is,

42 $$ \Gamma(v/2) = \int_{0}^{\infty} x^{v/2-1}e^{-x} dx.  $$

When we set the significance level to *α*, our proposed RandChiDist rejects *H*_0_ if the *χ*^2^ value calculated using Eq. (1), with the addition of Laplace noise and the scale *Δ*_*R*_/*ε*, is greater than or equal to *α*, as calculated by solving the following equation with regard to *α*;

43 $$ \int_{x=t}^{\infty} g_{v, \Delta, \epsilon}(x)= \alpha.  $$

Lastly, we compare the *χ*^2^^∗^ value calculated using Eq. (37) to the *t* value calculated using Eq. (43). When *χ*^2^^∗^ is greater than or equal to *t*, RandChiDist outputs “reject the null hypothesis *H*_0_,” and otherwise outputs “fail to reject the null hypothesis *H*_0_.”

Algorithm 1 shows the overall RandChiDist algorithm.


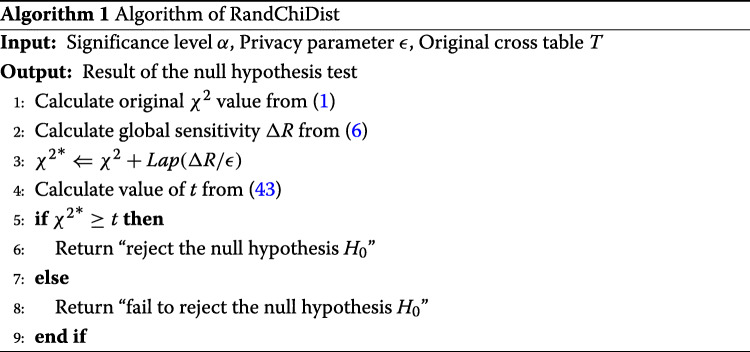


If we want an anonymized version of the *p* value, RandChiDist calculates and outputs

44 $$ \int_{x={\chi^{2}}^{*}}^{\infty} g_{v, \Delta, \epsilon}(x).   $$

The data analysis can thus conduct a *χ*^2^ hypothesis test using an arbitrary *α* by comparing Expression (44) and *α*.

### Complexity analysis

Calculating original *χ*^2^ yields a computational complexity of *O*(*I*×*J*). Calculating global sensitivity *ΔR* requires finding the largest value and the second largest value of *m*_*i*_(*i*=1,…,*I*); therefore, the computational complexity is *O*(*I*). Calculating (43) and (44) requires the calculation of an integration. For example, Monte Carlo integration can be adopted to calculate an integration. The computational complexity of Monte Carlo integration is not influenced by the cross table. There are numerous Monte Carlo integration methods that can be calculated extremely fast [55].

Therefore, the computational complexity of the proposed algorithm is *O*(*I*×*J*+*M*), where *M* denotes the computational complexity of calculating an integration.

## Evaluation

We compared RandChiDist, RandCell, MCIndep, and LocalExpIND as described in “Related work” section.

LocalExpIND was proposed especially for local privacy; therefore, LocalExpIND can be used for more scenarios than RandChiDist. Thus, the local model of privacy is another avenue for future exploration.

Moreover, to clarify the contribution of calculating the private *χ*^2^ distribution table’s value (proposed in “Differentially private hypothesis testing” section), we also compared a method that uses the global sensitivity *Δ*_*R*_ calculated using Eq. (6) that does not use the private *χ*^2^ distribution table’s value calculated using Eq. (24). We refer to this method as RandChi, which is also proposed in this paper.

The source code for the RandChi and RandChiDist methods can be obtained from https://uecdisk.cc.uec.ac.jp/index.php/s/pic3T9GEp03qy6y.

We should use Bonferroni’s corrected threshold when conducting multiple *χ*^2^ testing [56]. In this paper, we conducted many *χ*^2^ tests; however, we consider each to be independent. Thus, Bonferroni’s corrected threshold was not used in this paper to compare the performance among our proposed methods and methods from existing studies for independent *χ*^2^ testing. This paper shows the average results of each independent *χ*^2^ test. Additionally, previous studies of privacy-preserving *χ*^2^ testing, such as [7, 8, 27–29, 44, 45], did not use the Bonferroni’s corrected threshold.

We varied the values of *n* from 100 to 900, *α* from 0.005 to 0.05, and *ε* from 0.01 to 10. We set the parameters of MCIndep the same as in [8].

### Significance results

**Fig. 2:**
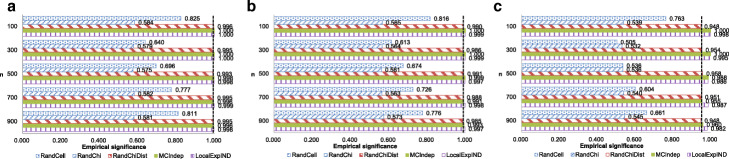
Significance results based on 2×2 contingency tables. The dashed lines represent 1−*α*. **a** Results with *ε*=0.1,*α*=0.005. **b** Results with *ε*=0.1,*α*=0.01. **c** Results with *ε*=0.1,*α*=0.05

We first evaluated the significance to confirm that RandChiDist guarantees a significance of at least 1−*α*. We randomly generated 2×2 contingency tables based on a multinomial distribution with probabilities of (0.25, 0.25, 0.25, 0.25) 1,000 times. Each time, we evaluated whether each method correctly output “fail to reject the null hypothesis *H*_0_.” Figure 2 shows the results with an *ε* value of 0.1. The significance of each method should be approximately 1−*α*.

The significance levels of RandChiDist, MCIndep, and LocalExpIND were controlled around 1−*α* for any *n*, *ε*, and *α* values. In contrast, RandCell and RandChi had significance values much less than 1−*α* when *ε* was less than 1.

**Fig. 3:**
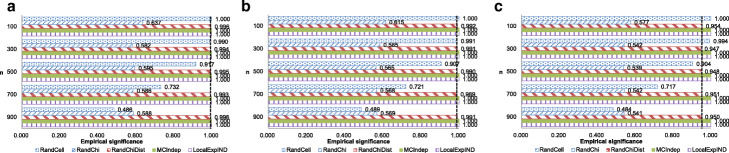
Significance results based on 4×4 contingency tables. The dashed lines represent 1−*α*. **a** Results with *ε*=0.1,*α*=0.005. **b** Results with *ε*=0.1,*α*=0.01. **c** Results with *ε*=0.1,*α*=0.05

We conducted the same experiments for randomly generated 4×4 contingency tables based on a multinomial distribution with probabilities of 1/16,…,1/16. Figure 3 shows the results with *ε*=0.1. As with the 2×2 contingency tables, the significance values of RandChiDist, MCIndep, and LocalExpIND were approximately 1−*α*. In contrast, RandCell and RandChi significance values were less than 1−*α*, especially when *ε* was small.

RandCell adds a Laplace noise to each cell. The probability that at least one Laplace noise becomes very large increases when the number of cells is large. Therefore, RandCell has many false positives (i.e., significance results are small) when contingency tables are large. On the other hand, if the Laplace noise is a large negative value, the cell value with the noise could be less than five (or negative). In this case, RandCell fails to reject the null hypothesis based on the rule of thumb. Therefore, RandCell’s results of 4 ×4 tables are smaller than those of 2 ×2 tables only when *n* is large.

RandChi’s significance results do not vary greatly by the table size or *n*. This is because the global sensitivity calculated from Eq. 7 also does not vary greatly by the table size or *n*.

### Power results

**Fig. 4:**
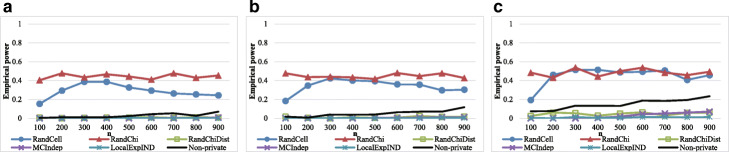
Empirical power results with 2×2 Contingency tables generated with probabilities of (0.25+0.01,0.25−0.01,0.25−0.01,0.25+0.01). **a** Results with *ε*=0.1,*α*=0.005. **b** Results with *ε*=0.1,*α*=0.01. **c** Results with *ε*=0.1,*α*=0.05

**Fig. 5:**
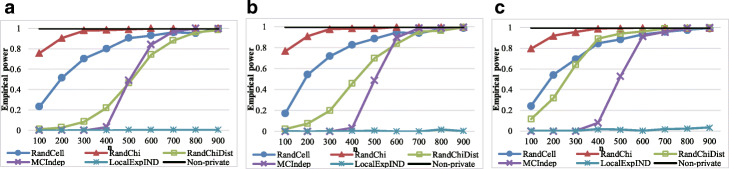
Empirical power results with 2×2 Contingency tables generated with probabilities of (0.25+0.15,0.25−0.15,0.25−0.15,0.25+0.15). **a** Results with *ε*=0.1,*α*=0.005. **b** Results with *ε*=0.1,*α*=0.01. **c** Results with *ε*=0.1,*α*=0.05

**Fig. 6:**
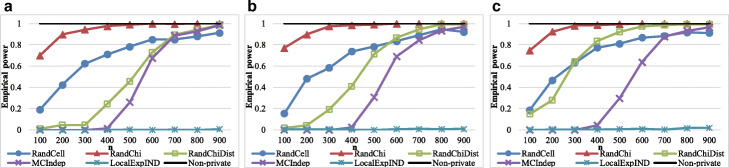
Empirical power results with 2×2 Contingency tables generated with probabilities of (0.3+0.15,0.3−0.15,0.2−0.15,0.2+0.15). **a** Results with *ε*=0.1,*α*=0.005. **b** Results with *ε*=0.1,*α*=0.01. **c** Results with *ε*=0.1,*α*=0.05

**Fig. 7:**
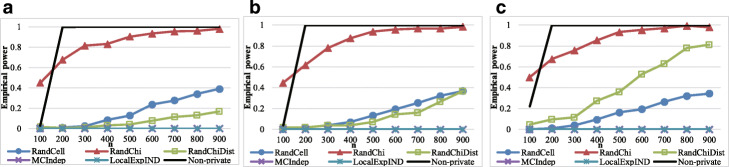
Empirical power results with 3×4 Contingency tables generated with probabilities of (1/12+0.07,1/12−0.07,1/12,1/12,1/12−0.07,1/12+0.07,1/12,…,1/12). **a** Results with *ε*=0.1,*α*=0.005. **b** Results with *ε*=0.1,*α*=0.01. **c** *ε*=0.1,*α*=0.05

We then evaluated each method’s power. The values of parameters *α*,*ε*, and *n* were identical to those in the significance experiments; however, we randomly generated 2×2 contingency tables based on a multinomial distribution with probabilities of (0.25+0.01,0.25−0.01,0.25−0.01,0.25+0.01) and (0.25+0.15,0.25−0.15,0.25−0.15,0.25+0.15). We also used another probability set (0.3+0.15,0.3−0.15,0.2−0.15,0.2+0.15) to determine whether RandChiDist can be applied to unbalanced tables. Moreover, we randomly generated 3×4 contingency tables based on a multinomial distribution with probabilities of (1/12+0.07,1/12−0.07,1/12,1/12,1/12−0.07,1/12+0.07,1/12,…,1/12). Each time, we evaluated whether each method correctly output “reject the null hypothesis *H*_0_.” Figures 4, 5, 6, and 7 show the results for *ε*=0.1.

In the experiment on a multinomial distribution with probabilities of (0.25+0.01,0.25−0.01,0.25−0.01,0.25+0.01), the empirical power of Non-private, which does not consider privacy at all, is very low, which is approximately from 0 to 0.2. Hence, all privacy-preserving algorithms that can control Type I errors do not realize high empirical power, although RandChiDist, which we proposed, is just slightly better than the other algorithms.

In the experiments on other multinomial distributions, MCIndep has relatively low empirical power. MCIndep generated many contingency tables from its algorithm based on the original contingency table. MCIndep quickly outputs “fail to reject *H*_0_” when at least one cell in the generated contingency tables has a value of less than five. Therefore, even if all the target contingency table’s cells have values greater than five, MCIndep is likely to output “fail to reject *H*_0_” if several values are close to 5 (for example a value of 10).

In contrast, RandCell and RandChi both achieved high empirical power at the expense of empirical significance. The empirical power of MCIndep is high when there are many samples and the data are uniformly distributed. RandChiDist achieved higher empirical power with fewer samples than MCIndep while also achieving empirical significance.

In hypothesis testing that includes *χ*^2^ testing, we should avoid Type I errors (i.e., false positives). In general, we adjust the Type I error probability by the value of *α* (e.g., 0.05). Even if the empirical power is high, the algorithm is of no use if the empirical significance is less than 1- *α*. The empirical power of RandCell and RandChi is greater than that of RandChiDist; however, RandCell and RandChi have empirical significance values much than 1- *α*. That is, they cannot control Type I errors (false positives) in many cases. Therefore, we can conclude that RandChiDist outperforms RandCell and RandChi. Among RandChiDist, MCIndep, and LocalExpIND, which can control Type I errors, RandChiDist has the highest power.

### Results of real datasets

We used two genomic datasets^1^. The first dataset is the Human Genome Diversity Project genotype dataset (HGDP) used by Conrad [57], which consists of 2,834 SNPs and has 1,244 records after the records in which unknown values are eliminated. The other is the International Haplotype Map Project genotype dataset (HapMap) used by [58], which consists of 1,853 SNPs and has 420 complete records.

We randomly generated contingency tables for linkage disequilibrium analysis for each dataset and set the numbers of columns and rows to four. Following the “rule of thumb,” if any values of the created contingency table are less than five, we re-created another contingency table and then conducted normal *χ*^2^ testing on the original contingency tables. We then carried out the privacy-preserving methods. We generated contingency tables and conducted *χ*^2^ testing 100 times, and then calculated the mean results of false positive and false negative rates.

**Fig. 8:**
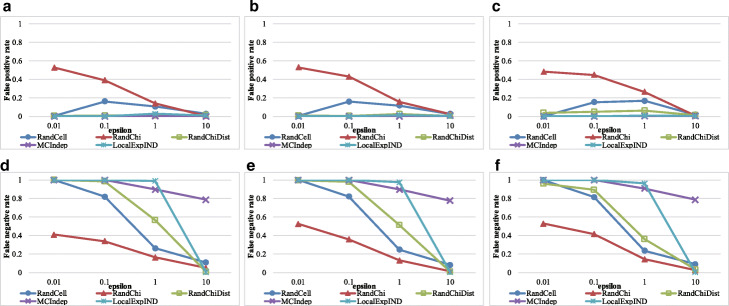
Results of the HGDP genotype datasets. **a** Results with False Positive Rate (*α*=0.005). **b** Results with False Positive Rate (*α*=0.01). **c** False Positive Rate (*α*=0.05). **d** False Negative Rate (*α*=0.005). **e** False Negative Rate (*α*=0.01). **f** False Negative Rate (*α*=0.05)

**Fig. 9:**
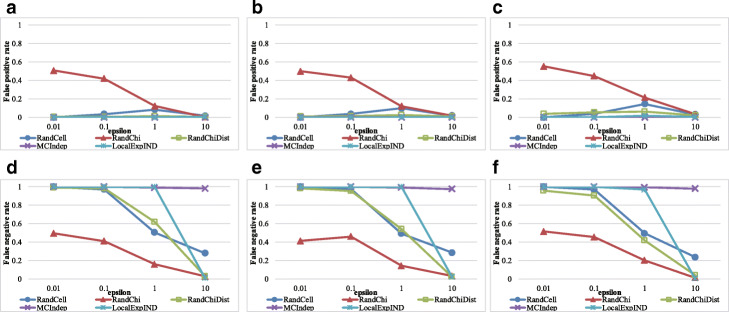
Results of the HapMap genotype datasets. **a** Results with False Positive Rate (*α*=0.005). **b** False Positive Rate (*α*=0.01). **c** False Positive Rate (*α*=0.05). **d** False Negative Rate (*α*=0.005). **e** False Negative Rate (*α*=0.01). **f** False Negative Rate (*α*=0.05)

The results for the HGDP genotype and HapMap genotype datasets are shown in Figs. 8 and 9, respectively. RandChiDist outperformed MCIndep and LocalExpIND for most of the parameter settings used in this paper.

## Discussion

According to the evaluation results, RandCell and RandChi could not control the ratio of Type I errors—that is, they caused a lot of false positives. On the contrary, RandChiDist, MCIndep, and LocalExpIND could control the ratio of Type I errors. RandChiDist achieved the least number of Type II errors among RandChiDist, MCIndep, and LocalExpIND. When testing a hypothesis, data analyzers determine the significance level *α* (i.e., the ratio of Type I errors) ahead of time. That is, they reject a true null hypothesis with a probability no greater than *α*. A high false positive rate means that a true null hypothesis is rejected with a probability greater than *α*, which leads to the false interpretation of datasets. Therefore, if we want to avoid such false interpretations, RandChiDist is the preferred method.

There are several approaches for non-private *χ*^2^ testing. The simplest approach is shown in “*χ*^2^ hypothesis test of Independence” section. RandChi and RandChiDist calculate the global sensitivity of the *χ*^2^ value of the simplest chi-squared testing and adds noise based on the global sensitivity to the *χ*^2^ value. Thus, the added noise is minimized following the Laplace mechanism theorem (Theorem 1).

In contrast, RandCell calculates the global sensitivity of each value of each cell and adds noise to each value. The summation of added noises thus become very large. MCIndep takes another approach for calculating non-private *χ*^2^ testing, as shown in “Related work” section. MCIndep first estimates the parameters of the underlying multinomial distribution generating the samples. By the estimated the multinomial distribution, MCIndep generates more than 1/*α* contingency tables. When the number of samples is small, the estimated parameters of the underlying multinomial distribution have low accuracy. Because of this low accuracy estimation, MCIndep could have low performance when the number of samples is small. LocalExpIND assumes that each piece data is anonymized for each person and that there is no trusted entity. Because noise is added to each data point, the amount of the total noise becomes large.

Sharpe claimed that if we can avoid *χ*^2^ hypothesis testing for contingency tables larger than 2 ×2, doing so is desirable [59]. However, he showed an understanding that in some cases we could not avoid this and also reported that approximately 30% of *χ*^2^ tests are conducted for contingency tables larger than 2 ×2. This is based on his survey of journals published by the American Psychological Association for 2012, 2013, and early 2014. *χ*^2^ hypothesis testing has been widely used for GWAS as well as many other personal databases [38–40]. Moreover, some studies [38–40] have considered contingency tables larger than 2 ×3. Therefore, we consider the application of *ε*-differential privacy to *χ*^2^ hypothesis testing for contingency tables larger than 2 ×3 to be an important issue.

Our proposed method can be used not only GWAS but also other private data analysis for small samples. For example, the characteristics of COVID-19 patients (*n*=403) (the number of died patients was 100 and the number of recovered patients was 303) were analyzed by *χ*^2^ test with *α* being 0.05 [60]. Poyiadi et al. analyzed the COVID-19 with acute pulmonary embolism and the COVID-19 without acute pulmonary embolism [61]. The number of patients was *n*=328. They conducted *χ*^2^ test with *α* being 0.05. The influence on sexual activity for COVID-19 was analyzed by Jacob et al. [62]. The number of samples was 868. As these studies show, there is a high need for testing with a small sample size. In particular, it is difficult to collect a large number of samples when there is a need for rapid analysis for a new disease such as COVID-19.

We assume that the data holder publishes *m*_*i*_ as well as the differentially private chi-square value. In general, the information of *m*_*i*_ and a sample size is necessary to interpret a chi-square value accurately [63]. For example, even if in the case of trivial differences between two datasets, a very small chi-square value is obtained when every *m*_*i*_ is very large [64]. Therefore, *m*_*i*_ is very useful information for data analysts.

Publishing *m*_*i*_ also provides several other types of information. For example, we know that *O*_*i*,*j*_ for all *j* are less than or equal to *m*_*i*_. However, we cannot know each value of *O*_*i*,*j*_, and we cannot know which value is greater (*O*_*i*,*j*_ or $\phantom {\dot {i}\!}O_{i,j'}$) for any *j* or *j*^′^, even if we know *m*_*i*_ and the (differentially private) chi-square value. Our proposed algorithm can protect chi-square values based on differential privacy, and we can ensure that it is impossible to reconstruct the original cross table. To the best of our knowledge, no researchers have claimed that publishing *m*_*i*_ could cause privacy issues.

## Conclusion

*χ*^2^ testing is widely used in GWAS and other types of data analysis. We proposed the RandChiDist method, which anonymizes the *χ*^2^ value of contingency tables. If we have a lot of samples for data analysis, it is easy to conduct statistical analysis precisely. However, obtaining highly sensitive data is quite difficult due to privacy reasons.Existing methods on privacy-preserving *χ*^2^ testing such as MCIndep are a better choice when the number of samples *n* is large; however, we demonstrated that RandChiDist outperforms existing methods when *n* is small.

Future work will include evaluating other relevant datasets. We also plan to apply our method to other hypothesis testing methods such as Student’s *t*-test and Fisher’s exact test.

## Data Availability

All data generated or analysed during this study are included in this published article.

## Footnotes

1 https://web.stanford.edu/group/rosenberglab/hgdpsnpDownload.html(accessed May 26, 2017)
